# Selected Risk Nutritional Factors for Chemotherapy-Induced Polyneuropathy

**DOI:** 10.3390/nu9060535

**Published:** 2017-05-25

**Authors:** Jiri Grim, Alena Ticha, Radomir Hyspler, Martin Valis, Zdenek Zadak

**Affiliations:** 1 Department of Oncology and Radiotherapy, University Hospital Hradec Kralove, 50005 Hradec Kralove, Czech; grimj@lfhk.cuni.cz; 2 Department of Research and Development, University Hospital Hradec Kralove, 50005 Hradec Kralove, Czech; rhyspler@lfhk.cuni.cz (R.H.); zdenek.zadak@fnhk.cz (Z.Z.); 3 Institute of Clinical Biochemistry and Diagnostics, University Hospital Hradec Kralove, 50005 Hradec Kralove, Czech; 4 Department of Neurology, University Hospital Hradec Kralove, 50005 Hradec Kralove, Czech; martin.valis@fnhk.cz

**Keywords:** vitamin B1, vitamin B6, vitamin D, fatty acids, polyneuropathy, chemotherapy

## Abstract

The present study seeks to identify the nutritional risk factors involved in the development of neuropathies induced by chemotherapeutic treatments. Unlike the gastrointestinal or hematological adverse effects of chemotherapy there is no protective treatment strategy for polyneuropathy. The aim of this study was to find possible deficiencies in nutritional factors, which can be used for supplementation in the future for prevention of chemotherapy-induced neuropathy development. We analyzed 70 patients undergoing paclitaxel chemotherapy and evaluated the risk factors involved in chemotherapy-induced peripheral neuropathy (CIPN). Several risk factors were considered in the development of CIPN, including deficiency of vitamin B1, B6, and D and fatty acids. The occurrence of CIPN complication in 60% cases was observed. We found significant differences in vitamin D and saturated fatty acid concentration. Vitamin D levels in the group without CIPN were estimated to be 38.2 (24.95, 47.63) nmol/L, whereas in the group with CIPN it was determined to be 25.6 (19.7, 32.55) nmol/L, *p* = 0.008. The level of total saturated fatty acids in the group without CIPN was of 32.613 Area % (31.322; 36.262), whereas in the group with CIPN it was of 34.209 Area % (32.86; 39.386), *p* = 0.01. The obtained results suggest a diet lower in saturated fatty acid content during chemotherapy. The most significant finding was that supplementation of vitamin D before chemotherapy could be an efficient neuroprotective in CIPN prophylaxis, as significantly lower levels 25OH derivative of vitamin D were observed in the CIPN group throughout the study period.

## 1. Introduction

Chemotherapy-induced peripheral neuropathy (CIPN) is an undesirable side effect and a limiting factor of systemic chemotherapeutic treatments. Unlike gastrointestinal or hematological toxicity, no protective treatment strategy has yet been implemented to prevent CIPN occurrence. To date, there are no specific markers predicting the potential development of polyneuropathy after the application of neurotoxic chemotherapy.

It is a known fact that antineoplastic agents can lead to degenerative changes in peripheral sensory and, with less frequency, motor nerve fibers, resulting in a distorted perception of cold, heat, and pain that in turn cause general weakness and balance problems. Overall, sensory fibers are more vulnerable to damage in comparison to motor nerves [1] due to the hematoencephalic barrier preventing the penetration of cytotoxic drugs into the central nervous system (CNS), where the body of motor nerve neurons is located, protected against the effects of these drugs [2,3]. However, the primary sensory and autonomic neurons are located outside of the central nervous system, unprotected by the hematoencephalic barrier [1]. The capillaries of these neurons have fenestrated walls, enabling the relatively easy transfer of drug molecules between the blood capillaries and the extracellular space of ganglia. The long axons of peripheral nerves are very sensitive to damage induced by energy metabolism or to compromised structures serving the axoplasmic transport. Microtubule damage by taxanes and vinca alkaloids in particular results in axoplasmic transport failure [1].

Often, drugs that inhibit the mitochondrial activity of cancer cells also affect the axoplasmic transport of neurons, and chemotherapeutic agents can, in addition, activate the processes of programmed cell death [1,2]. Although neurons are highly organized cells, they lack the capacity to divide further and are therefore unable to self-renew [3]. For this reason, patients often complain of numbness, tingling, or burning sensations. A patient may also perceive pain stimuli known as allodynia, e.g., after cisplatin administration, some people perceive contact with cold surfaces as painful. In addition, patients sometimes present a reduced perception for vibrations, and reduced or absent reflexes [4,5,6]. Sensory symptoms often begin in the tips of fingers or toes, and can spread in a proximal manner in a stocking or glove pattern; more rarely, some patients develop a feeling of weakness [1] (Table 1). After taxanes or vinca alkaloid-based chemotherapy, most toxic neuropathies include disabilities in long and sensitive fibers [1]; on the other hand, platinum complexes cause rather sensitive gangliopathies [1]. Patients treated with taxanes sometimes present balance and dexterity issues (short walk tests require more time) [6].

After chemotherapy, patients have reported stumbling and motor problems when walking. They also complain about a deteriorated perception of the surface on which they are walking and do not perceive it as flat but rather as if walking on a stony path or on gravel; they can also feel the need to hold the railing when going up or down flights of stairs. In addition, patients with painful (sensory form) CIPN had more trouble with the fine motor function of their hands [1], which can be observed in patients’ poor writing skills or in their difficulty typing [4,5,6]. The severity of the resulting neuropathy commonly depends upon the dose of the administered drug; however, neuropathy progression also stops with the conclusion of the chemotherapeutic course [9]. Platinum complexes are the exception to this rule, and the progression of sensitive disorders continues even after a period of several months following the discontinuation of the drug (“coasting”).

The synergic exposure to several neurotoxic drugs may be followed with the development of severe neuropathy [4]. In addition to chemotherapy, a previously existing inflammatory neuropathy condition, particularly of a diabetic nature, also predisposes the development of CIPN in the treated patient [11].

Unlike the gastrointestinal or hematological adverse effects of chemotherapy, there is no protective treatment strategy for polyneuropathy. In addition, nutritional factors known to exacerbate the potential damage to the peripheral nervous system were chosen based on previous reports, i.e., omega-3 fatty acids (eicosapentaenoic acid (EPA) and docosahexaenoic acid(DHA)) [12,13] and vitamin B1 and B6.

Malnutrition and vitamin B deficiency (vitamin B1, B6, and B12, in particular) are known mechanisms in the emergence of polyneuropathies [14]. This same deficiency could be assumed in patients undergoing chemotherapy [14]. Additionally, the patients undergoing oncology treatment could present low levels of omega fatty acids (essential cis-polyunsaturated fatty acids omega-3 and omega-6) [13]. 

On the other hand, vitamin D deficiency could play a role in the repair of axonal damage [8]; therefore, plasmatic 25-hydroxy vitamin D level was monitored in this study as a possible prediction factor of chemotherapy-induced polyneuropathy.

The aim of this study was to find possible deficiencies in nutritional factors, which can be used for supplementation in the future for prevention of chemotherapy-induced neuropathy development. Based on previous reports, the following markers were selected: vitamin B1 and B6, omega 3 fatty acids (EPA and DHA), and vitamin D. Our study suggests that monitoring these analytes in patients undergoing chemotherapy could establish a direct correlation with the appearance of polyneuropathy.

## 2. Patients and Methods

A monocentric and prospective evaluation was performed as an open case–control study in patients undergoing paclitaxel chemotherapy. The study focused in assessing the prediction potential and availability of plasma biomarkers in the development of polyneuropathy complications. The study included patients (breast carcinoma) from the Clinic of Oncology and Radiotherapy undergoing post-operative (adjuvant), preoperative (neo-adjuvant), or palliative chemotherapy based on 80 mg/m^2^ paclitaxel on a weekly basis (12 cycles). After signing the informed consent, the patients were examined using a standardized Michigan questionnaire evaluating the absence or presence of a neuropathy, or eventually the valuation of its seriousness. A sample of 6 mL of blood (EDTA as anticoagulant, BD Vacutainer, Leicestershire, UK) was taken from each volunteer for further biochemical analysis. This procedure was repeated in Week 4 and Week 12 of chemotherapy (4th and 12th cycles of chemotherapy with paclitaxel). All experimental procedures were approved by the Ethics Committee (Reference number 201511 S09P) according to the Declaration of Helsinki (June 1964 and later amended). Eligible patients were properly informed of the study’s aim and methods in both verbal and written form, and a willing and informed consent was obtained. The study group is described in Table 2. No neuropathy was diagnosed in patients with diabetes before initiation of chemotherapy regimes. Patient suffering from diabetic polyneuropathy usually do not follow chemotherapy by taxanes but only the chemotherapy of antracyclines and cyclophosphamide [15,16].

Each of the studied analytes considered as potential predictors of neuropathy were compared with the clinical state of the patient and correlated with the acquired values. The data was evaluated by one-way ANOVA and repeated measures one-way ANOVA using the software Sigmastat (Systat, San Jose, CA, USA). The data is presented as mean ± SD or median (25%; 75%) and the difference was considered statistically significant when *p* ≤ 0.05. Receiver operating characteristic (ROC) was determined using the software Analyse-it for MS Excel (Analyse-it Software, Ltd., Leeds, UK).

The clinical evaluation of the patients was determined using the Michigan questionnaire, a Michigan neuropathy screening instrument, patient version (A) and physical assessment (B); MNSI, University of Michigan, 2000 [17]. The scores were calculated as ratio/total score (interval from 0 to 1) and are presented in Table 3. The evaluation of the patients was performed (a) before chemotherapy (Evaluation 0), (b) during chemotherapy (Week 4; Evaluation 1), and (c) at the end of chemotherapy (Week 12, Evaluation 2). 

Plasma levels of vitamin B1 and B6 were analyzed using the ELISA kit for Vitamin B12 and B6 (Cloud-Clone Corp., Katy, TX, USA) according to the manufacturer’s instructions. Vitamin D levels were analyzed by Liaison XL (DiaSorin, Saluggia, Italy). The concentration of fatty acids in plasma was analyzed using gas chromatography with a flame ionization detector (Dani Master, DANI Instruments S.p.A., Cologno, Italy). Following its extraction from plasma by toluen-acetylchloride and derivatization using methanol, chromatographic separation was performed in an RTX-2330 column (60 m, 0.25 mm ID, 0.2 um df, Restek, Bellefonte, PA, USA). Determined fatty acid: C12:0 (lauric acid), C14:0 (myristic acid), C16:0 (palmitic acid), C16:1 (palmitoelic acid), C18:0 (steariuc acid), C18:1 (oleic acid), C18:2 n6 (linoleic acid), C18:3 n6 (gamalinolenic acid), C18:3 n3 (alfalinolenic acid), C20:3 n6 (dihomogamalinolenic acid), C20:4 n6 (arachidonic acid), C22:6 n3 (docosahexaenoic acid), tSFA (total saturated fatty acid), n6 (sum of omega 6 fatty acids), n3 (sum of omega 3 fatty acids), AA/EPA (arachidonic and eicosapentaenoic acid ratio), tPUFA (total polyunsaturated fatty acid) are presented as Area %.

CIPN absence was identified and scored as 0 (CIPN 0) during the evaluation; the occurrence of any symptoms was considered as indicative of CIPN presence and scored accordingly as 1 (CIPN 1) (Table 4 and Table 5).

## 3. Results

**Figure 1:**
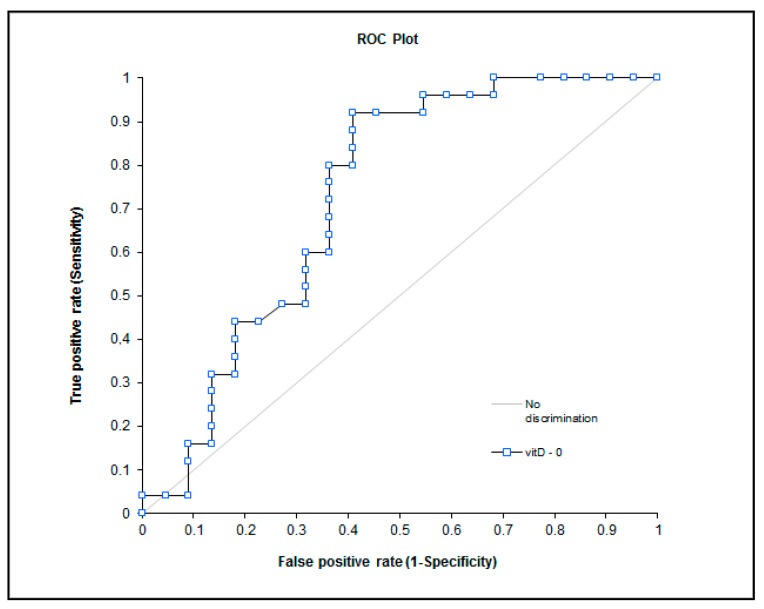
ROC curve—vitamin D (Area 0.73; *p* = 0.0023), cutoff value 34 nmol/L—sensitivity 80%, specificity 65%.

**Figure 2:**
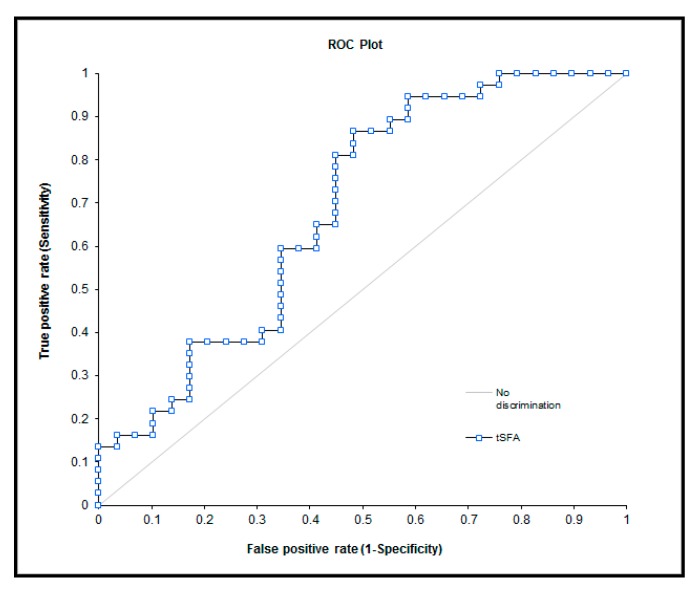
ROC curve—selected fatty acid—total saturated fatty acids (area 0.68, *p* = 0.0036), omega 7 class—palmitoopleic acid—C16:1 (area 0.65, *p* = 0.01) and sum of omega 3 fatty acid (area 0.57, *p* = 0.152).

The biochemical values obtained from the evaluation of the groups with and without CIPN prior to chemotherapy were found significant (Table 4). The obtained biochemical results during observations of the study groups are presented in Table 5. The potential of these markers as predictors of polyneuropathy are presented in Figure 1 and Figure 2.

Prior to chemotherapeutic treatment, we observed statistically significant differences between the CIPN 1 and CIPN 0 groups concerning vitamin D deficiency, unsaturated fatty acids (C12:0, C14:0, C16:1), and total saturated fatty acids (tSFA). 

The polyneuropathy group (CIPN 1) presented a significant difference between the concentration of fatty acids before, and/or during, and/or the end of therapy, i.e., C12:0, C14:0, C16:0, C 18:0, C18:2 n6, C20:3 n6, C20:4 n6, C20:5 n3, tSFA, n6, n3, AA/EPA (ratio arachidonic acid and eicosapentaenoic acid), and total polyunsaturated fatty acids (tPUFA). Additionally, vitamin D concentration was found in a significantly lower concentration during treatment. 

The group without polyneuropathy (CIPN 0) also showed a significant difference between the concentration of fatty acids before, during, and at the end of chemotherapeutic treatment, i.e., C14:0, C16:0, C18:3 n6, C20:3 n6, C20:4 n6, C22:6 n3, tSFA, n6, n3, and tPUFA. Likewise, vitamin D concentration decreased significantly during the treatment. However, with the exception of n3, the drop in the evaluated parameters was lower in the control group than in the CIPN 1 group. 

The analysis on polyneuropathy occurrence in cancer patients after chemotherapy, evaluated at Weeks 1, 4, and 14 post-treatment, reflects the worsening of symptoms induced by chemotherapeutic drugs. 

## 4. Discussion

We performed a prospective evaluation of risk factors involved in chemotherapy-induced polyneuropathy, although a complete description of chemotherapy-induced neuropathy risk and protective factors has yet to be defined. Our prospective trial addressed the clinical features of chemotherapy-induced neuropathy and the relationship between the severity of the pathology and the depletion of potential protective factors. The incidence of polyneuropathy is partially dependent on the frequency of given taxane-based or oxaliplatine-based medication.

Recapitulating previous studies, cisplatine and taxol induced the symptom onset of a pure sensory neuropathy, involving compromised sensitivity to pain, touch, and vibrations in most of our patients [1,4,5,6,12,14,18,19], rarely reporting a painful sensation. Our clinical results were also similar to data reported previously i.e., distal, symmetric neuropathy involving firstly the legs and secondly the arms in most cases [1,14,18,19]. Often, the symptoms of a sensory neuropathy, induced by oxaliplatine and taxol, were already obvious at the time of the second neurologic examination, after four initial courses of treatment. As in other studies, the manifestation and symptoms of motor and autonomic impairment were mostly absent in our patient population [1,18]. No patients suffered from autonomic impairment—no problems with voiding or stool in any patient.

The peripheral toxicity of oxaliplatine and taxol is seemingly correlated with the individual dose administered (single-dose toxicity) and with the total dose during chemotherapy, particularly where oxaliplatine is concerned [1,20]. However, a cumulative effect has not been reported by other authors so far [18]. The experimental design of our trial also failed to provide a definitive answer regarding the total-dose or single-dose toxicity correlation. 

The potential of diverse biochemical markers as CIPN prediction factors was based on previous studies [6,7,8]. Vitamin B1 and B6 deficiency have known effects in diabetic neuropathy. These vitamins were monitored in plasma, showing similar levels of vitamin B1 and B6 in between our study groups (Table 4 and Table 5).

The repletion of vitamin D before chemotherapy could be an efficient neuroprotective in CIPN prophylaxis, as significantly low levels 25OH derivative of vitamin D were observed in the CIPN group throughout the study period (Table 4 and Table 5). The obtained operating curve (Figure 1) presents a good area under the curve; however, it has a lower specificity at the cut off value of34 nmol/L. This observation could have been explained in a previous report [21], although we propose differences in the biological active form of vitamin D. The vitamin D binding protein is a precursor of the immunomodulating protein Gc-protein-derived macrophage activating factor that increases the activity of macrophages against tumors. Although the vitamin D binding protein has a bigger binding affinity for vitamin D in contrast to albumin, the bioavailable active form of vitamin D is the sum of free and albumin-bound vitamin D. We hypothesize that oncology patients present a lower concentration of serum albumin and a higher level of vitamin D binding protein. Therefore, the calculation of the bioavailable active form of vitamin D could prove useful in the prediction of CIPN. 

Fatty acids present in plasma are suitable markers of the nutritional status of patients. Although the dietary intake of saturated fatty acids could be a limited factor in nutrition, the sum of total saturated fatty acids (lauric and myristic acid) was significantly higher in the CIPN group (Table 4). Saturated fatty acids can activate the toll-like receptor 4 and are antagonists for the toll-like receptor 4/lipid-binding accessory protein MD2 complex; they are also capable of inducing the production of inflammatory cytokines and interferons [22]. These changes could explain the observed differences between both groups of patients. The observed changes in omega-7 (palmitoleic acid) content are probably caused by a different diet. The ROC curve (Figure 2) shows that the sum of saturated fatty acids cannot be used as a predictor of CIPN occurrence. For comparison, palmitoleic acid (omega 7 fatty acid class) and the sum of omega 3 fatty acid ROC curves were added to Figure 2. In all of these cases, the parameters of the ROC curve were found to be unsuitable as predictors of CIPN. Omega-3 levels in plasma were not found to be a suitable predictor factor in our study, contrary to other authors [7]. Based on our results, we suggest that a lower dietary intake of saturated fatty acids during chemotherapy can only be beneficial for the patients. 

## 5. Conclusions

In the present study, we found that vitamin D deficiency is involved in the development of chemotherapy-induced polyneuropathy. The total content of saturated fatty acids, along with the presence of lauric and myristic acids, was significantly higher in the group of CIPN patients. Regardless of this, the level of saturated fatty acids cannot be used as a predictor of CIPN occurrence per se. Although interesting, our preliminary results have yet to be verified in future clinical studies assessing vitamin D level in patients prior to chemotherapeutic treatment; such information would be useful in the selection of a more suitable alternative treatment.

## Figures and Tables

**Table 1:** Frequently used chemotherapeutic agents that have been associated with peripheral neuropathy, adjusted from [2,7,8,9,10].

Chemotherapy Agent	Incidence of CIPN	Sensory Symptoms	Motor Symptoms	Recovery
Taxane Class				
Paclitaxel (Taxol^®^)	50–70%	Mild distal loss of sensation to all modalities, feet greater than hands, painful paresthesias. Mild to moderate numbness, tingling, burning/stabbing pain of hands and feet are common, which can become severe with increased doses.	Weakness of distal muscles has been documented with high cumulative doses of paclitaxel and docetaxel.	Usually improves after treatment, but persistent symptoms in about 50% of patients one year later.
Docetaxel (Taxotere^®^)	40–60%			
Abraxane™	60–80%			
Vinca Alkaloid Class				
Vincristine (Onkovin^®^)	50–70% (1)	Distal sensory loss lower extremities, rarely affects upper extremities; vinblastine and vinorelbine are less neurotoxic; vincristine-rare mononeuropathies.	Weakness of distal muscles, decreased deep tendon reflexes, and foot drop have been noted with high doses.	Usually resolves within three months; may persist with vincristine.
Vinorelbine (Navelbine^®^)	20–30%			
Platinum Compounds				
Cisplatin (Platinol^®^)	40–60% (2, 3)	Distal, symmetric loss of sensation to all modalities, stocking glove distribution; painful paresthesias or numbness. Symptoms can become severe with high cumulative doses.	Weakness is rare but can occur with high doses of cisplatin and oxaliplatin.	Partial; may progress for several months after drug is discontinued.
Carboplatin (Paraplatin^®^)	2–8%			
Oxaliplatin (Eloxatin^®^)	70–80%			

CIPN: chemotherapy-induced peripheral neuropathy.

**Table 2:** Description of the study group.

Characteristic	Number of Cases, %, or Mean ± SD
Total number of patients	70
Sex	10% males; 90% females
Age (years)	56 ± 12.2
Body mass index (kg m^−2^)	27.2 ± 5.5
diabetes mellitus	9.5%
tumor presence	Non 66%; neo-adjuvant 14%; palliative 8%
CIPN complication	60%

CIPN—chemotherapy-induced peripheral neuropathy.

**Table 3:** Neuropathy evaluation by means of a Michigan questionnaire.

	Median 0 (25%; 75%)	Median 1 (25%; 75%)	Median 2 (25%; 75%)	*p* Value
Score doctor	0 (0; 0)	0.2 (0.175; 0.2)	0.3 (0.3; 0.4)	<0.05 *^,^**^,^***
Score patient	0 (0; 0)	0.133 (0.133; 0.117)	0.2 (0.2; 0.267)	<0.05 *^,^**^,^***

Significance * 0 vs. 1; ** 1 vs. 2; *** 0 vs. 1. Evaluation 0—before chemotherapy (Week 1), Evaluation 1—during chemotherapy (Week 4), Evaluation 2—after chemotherapy (Week 12).

**Table 4:** Biochemical values before chemotherapy in patients with (1) and without (0) CIPN.

Analyte	CIPN	Median	Mean ± SD	*p* Value
		(Interquartile Range)		
**Vitamin B1** (ng/mL)	0	10.1 (6.38; 21.6)	-	ns
	1	9.72 (6.67; 14.1)	-	
**Vitamin B6** (ng/mL)	0	6.65 (4.84; 14.3)	-	ns
	1	9.38 (4.6; 15.5)	-	
**Vitamin D** (nmol/L)	0	38.5 (24.95; 47.63)	-	**0.008**
	1	25.6 (19.7; 32.55)	-	
**C12:0** (Area %)	0	0.18 (0.11; 0.31)	-	**0.031**
	1	0.24 (0.18; 0.42)	-	
**C14:0** (Area %)	0	1.42 (1.02; 1.94)	-	**0.041**
	1	1.62 (1.34; 2.3)	-	
**C16:0** (Area %)	0	24.34 (23.06; 27.23)	-	ns
	1	24.96 (23.7; 29.25)	-	
**C16:1** (Area %)	0	-	2.42 ± 0.77	**0.03**
	1	-	2.96 ± 1.11	
**C18:0** (Area %)	0	-	7.03 ± 0.65	ns
	1	-	7.24 ± 0.69	
**C18:1** (Area %)	0	-	24.65 ± 3	ns
	1	-	24.19 ± 2.64	
**C18:2 n6** (Area %)	0	-	27.36 ± 3.9	ns
	1	-	26.19 ± 3.73	
**C18:3 n6** (Area %)	0	0.48 (0.26; 0.6)	-	ns
	1	0.48 (0.14; 0.6)	-	
**C18:3 n3** (Area %)	0	0.52 (0.43; 0.71)	-	ns
	1	0.52 (0.46; 0.68)	-	
**C20:3 n6** (Area %)	0	1.96 (1.46; 2.33)	-	ns
	1	1.89 (1.02; 2.3)	-	
**C20:4 n6** (Area %)	0	-	6.39 ± 1.47	ns
	1	-	6.12 ± 1.8	
**C20:5 n3** (Area %)	0	0.67 (0.4; 0.75)	-	ns
	1	0.66 (0.46; 0.81)	-	
**C22:6** (Area %)	0	-	1.32 ± 0.54	ns
	1	-	1.13 ± 0.62	
**tSFA**	0	32.61 (31.32; 36.26)	-	**0.01**
	1	34.21 (32.86; 39.39)	-	
**tMFA**	0	-	27.07 ± 3.3	ns
	1	-	27.14 ± 3.2	
**n6**	0	-	35.64± 4.22	ns
	1	-	34.02 ± 4.43	
**n3**	0	2.43 (1.97; 3.13)	-	ns
	1	2.35 (1.62; 3.12)	-	
**AA/EPA**	0	9.99 (7.72; 14.25)	-	ns
	1	9.6 (6.9; 11.96)	-	
**tPUFA**	0	-	38.4 ± 4.6	ns
		-	36.4 ± 4.9	

The data is presented as median (interquartile range—25th percentile; 75th percentile) or mean ± SD. CIPN—chemotherapy-induced peripheral neuropathy; ns—not significant; C12:0—lauric acid; C14:0—myristic acid; C16:0—palmitic acid; C16:1—palmitoelic acid; C18:0—steariuc acid; C18:1—oleic acid; C18:2 n6—linoleic acid; C18:3 n6—gamalinolenic acid; C18:3 n3—alfalinolenic acid; C20:3 n6—dihomogamalinolenic acid; C20:4 n6—arachidonic acid; C22:6 n3—docosahexaenoic acid; tSFA—total saturated fatty acid; n6—sum of omega 6 fatty acids; n3—sum of omega 3 fatty acids; AA/EPA—arachidonic and eicosapentaenoic acid ratio; tPUFA—total polyunsaturated fatty acid.

**Table 5:** Biochemical values observed in separate groups (0—without CIPN; 1—with CIPN) during sampling times, i.e., before (0), during (1), and at the end (2) of treatment.

Analyte	CIPN	Mean 0 ± SD	Mean 1 ± SD	Mean 2 ± SD	*p* Value
Vitamin B1 (ng/mL)	0	15.12 ± 11.8	14.43 ± 8.1	11.48 ± 5.6	ns
	1	11.5 ± 6.6	11.2 ± 5.4	9.3 ± 4	ns
Vitamin B6 (ng/mL)	0	10.93 ± 9.7	10.08 ± 5.06	8.77 ± 5.3	ns
	1	11.85 ± 9.7	9.64 ± 6.9	12.74 ± 9.9	ns
Vitamin D (nmol/L)	0	38.08 ± 15.6	37.44 ± 19.9	42.33 ± 9.6	<0.05 *
	1	26.94 ± 8.5	24.28 ± 8.9	31.4 ± 10.4	<0.001 *^,#^
C12:0 (Area %)	0	0.28 ± 0.18	0.26 ± 0.14	0.41 ± 0.23	<0.05 *^,#^
	1	0.36 ± 0.23	0.38 ± 0.2	0.34 ± 0.16	ns
C14:0 (Area %)	0	1.53 ± 0.6	1.95 ± 0.9	2.5 ± 0.8	<0.05 *
	1	1.94 ± 0.9	2.32 ± 0.9	2.1 ± 0.85	<0.05 *^,#^
C16:0 (Area %)	0	25.23 ± 2.7	26.27 ± 3.6	29.44 ± 2.9	<0.05 ^#,^**
	1	26.49 ± 3.41	28.78 ± 3.6	29.45 ± 4.1	<0.001 *^,#,^**
C16:1 (Area %)	0	2.42 ± 0.7	2.8 ± 0.9	2.89 ± 0.8	ns
	1	2.96 ± 1.1	3.2 ± 0.84	2.96 ± 1.1	ns
C18:0 (Area %)	0	7.03 ± 0.65	7 ± 0.72	6.76 ± 0.73	ns
	1	7.24 ± 0.7	6.95 ± 0.65	7.1 ± 0.16	<0.001 *^,^**
C18:1 (Area %)	0	24.65 ± 3	23.7 ± 3.4	23.78 ± 2.5	ns
	1	24.18 ± 2.6	24.34 ± 2.7	23.22 ± 2.6	ns
C18:2 n6 (Area %)	0	27.36 ± 3.9	27.11 ± 3.4	26.2 ± 3.76	<0.05 *^,#^
	1	26.19 ± 3.7	25.2 ± 4	26.44 ± 4.1	ns
C18:3 n6 (Area %)	0	0.46 ± 0.22	0.4 ± 0.25	0.17 ± 0.11	<0.05 *
	1	0.42 ± 0.27	0.27 ± 0.23	0.23 ± 0.2	<0.001 *^,#,^**
C18:3 n3 (Area %)	0	0.82 ± 0.65	1.02 ± 0.76	0.82 ± 0.62	ns
	1	0.57 ± 0.2	0.54 ± 0.18	0.55 ± 0.2	ns
C20:3 n6 (Area %)	0	1.89 ± 0.54	1.62 ± 0.56	1.14 ± 0.34	<0.05 *
	1	1.72 ± 0.7	1.41 ± 0.63	1.24 ± 0.56	<0.001 *^,#,^**
C20:4 n6 (Area %)	0	6.39 ± 1.47	5.89 ± 1.89	4.59 ± 1.3	<0.05 *
	1	6.12 ± 1.8	5.22 ± 1.7	4.95 ± 1.5	<0.001 *^,#,^**
C20:5 n3 (Area %)	0	0.62 ± 0.3	0.86 ± 0.56	0.74 ± 0.65	ns
	1	0.68 ± 0.3	0.6 ± 0.28	0.71 ± 0.4	ns
C22:6 (Area %)	0	1.32 ± 0.54	1.11 ± 0.6	0.56 ± 0.3	<0.001 *^,#^
	1	1.13 ± 0.6	0.78 ± 0.6	0.72 ± 0.6	<0.001 *^,#,^**
tSFA	0	34.1 ± 3.5	35.5 ± 4.15	39.11 ± 3.33	<0.05 *^,#^
	1	36 ± 4.1	38.44 ± 4.4	38.98 ± 4.5	<0.001 *^,#,^**
tMFA	0	27.1 ± 3.3	26.52 ± 3.7	26.67 ± 2.64	ns
	1	27.14 ± 3.2	27.53 ± 3.1	26.2 ± 3	ns
n6	0	35.64 ± 4.2	34.61 ± 4.1	31.93 ± 3.62	<0.05 *^,#^
	1	34.02 ± 4.42	31.83 ± 4.7	32.63 ± 4.9	<0.001 *^,^**
n3	0	2.76 ± 1.45	2.99 ± 2.23	2.12 ± 1.84	<0.05 *
	1	2.38 ± 0.9	1.93 ± 0.98	1.97 ± 1.1	<0.001 *^,#,^**
AA/EPA	0	13.1 ± 8.5	8.15 ± 2.72	8.1 ± 3.56	ns
	1	9.92 ± 3.24	9.48 ± 3.15	8.44 ± 3.82	<0.05 *
tPUFA	0	38.4 ± 4.64	37.6 ± 5.5	34.05 ± 4	<0.001 *^,#^
	1	36.4 ± 4.9	33.75 ± 5	34.6 ± 5.4	<0.001 *^,#,^**

CIPN 0 and CIPN 1 subgroups were not compared but rather evaluated by themselves. CIPN—chemotherapy-induced peripheral neuropathy; ns—no significant; C12:0—lauric acid; C14:0—myristic acid; C16:0—palmitic acid; C16:1—palmitoelic acid; C18:0—steariuc acid; C18:1—oleic acid; C18:2 n6—linoleic acid; C18:3 n6—gamalinolenic acid; C18:3 n3—alfalinolenic acid; C20:3 n6—dihomogamalinolenic acid; C20:4 n6—arachidonic acid; C22:6 n3—docosahexaenoic acid; tSFA—total saturated fatty acid; n6—sum of omega 6 fatty acids; n3—sum of omega 3 fatty acids; AA/EPA—arachidonic and eicosapentaenoic acid ratio; tPUFA—total polyunsaturated fatty acid. Significant differences: * 0 vs. 1; ^#^ 1 vs. 2; ** 0 vs. 2.
